# Knowledge, attitudes, and practice of general practitioners toward community detection and management of mild cognitive impairment: a cross-sectional study in Shanghai, China

**DOI:** 10.1186/s12875-022-01716-9

**Published:** 2022-05-11

**Authors:** Yuan Lu, Chaojie Liu, Sally Fawkes, Zhaoxin Wang, Dehua Yu

**Affiliations:** 1 Department of General Practice, Yangpu Hospital, Tongji University School of Medicine, Shanghai, 200090 China; 2 School of Psychology and Public Health, La Trobe University, Melbourne, VIC 3086 Australia; 3 Tongji University School of Medicine, Shanghai, 200092 China; 4 Shanghai General Practice and Community Health Development Research Center, Shanghai, 200090 China; 5 School of Public Health, Shanghai Jiaotong University School of Medicine, Shanghai, 200025 China

**Keywords:** Mild cognitive impairment, KAP, General practitioners, China

## Abstract

**Background:**

General practitioners (GPs) are in a unique position for community detection and management of mild cognitive impairment (MCI). However, adequate knowledge, attitudes, and practice (KAP) are prerequisites for fulfilling such a role. This study aims to assess the MCI-related KAP of GPs in Shanghai, China.

**Methods:**

An online survey was conducted on 1253 GPs who were recruited from 56 community health centres (CHCs) in Shanghai between April and May 2021. Knowledge (8 items), attitudes (13 items), and practice (11 items) were assessed using a scale endorsed by a panel of multidisciplinary experts. An average summed score was calculated and transformed into a score ranging from 0 to 100 for knowledge, attitudes, and practice, respectively. Adjusted odds ratios (AORs) were calculated for potential predictors of higher levels of KAP scores (with mean value as a cutoff point) through logistic modelling. The mediating role of attitudes on the association between knowledge and practice was tested using the PROCESS model 4 macro with 5000 bootstrap samples through linear regression modelling.

**Results:**

A total of 1253 GPs completed the questionnaire, with an average score of 54.51 ± 18.18, 57.31 ± 7.43, and 50.05 ± 19.80 for knowledge, attitudes, and practice, respectively. More than 12% of respondents scored zero in knowledge, 28.4% tended not to consider MCI as a disease, and 19.1% completely rejected MCI screening. Higher levels of knowledge were associated with more favourable attitudes toward community management of MCI (AOR = 1.974, *p* < 0.001). Higher compliance with practice guidelines was associated with both higher levels of knowledge (AOR = 1.426, *p* < 0.01) and more favourable attitudes (AOR = 2.095, *p* < 0.001). The association between knowledge and practice was partially mediated by attitudes (*p* < 0.001). Training was associated with higher levels of knowledge (AOR = 1.553, *p* < 0.01), while past experience in MCI management was associated with more favourable attitudes (AOR = 1.582, *p* < 0.05) and higher compliance with practice guidelines (AOR = 3.034, *p* < 0.001). MCI screening qualification was associated with higher compliance with practice guidelines (AOR = 2.162, *p* < 0.05), but less favourable attitudes (AOR = 0.452, *p* < 0.05).

**Conclusion:**

The MCI knowledge of GPs in Shanghai is low, and is associated with less favourable attitudes toward MCI management and low compliance with practice guidelines. Attitudes mediate the association between knowledge and practice. Training is a significant predictor of knowledge. Further studies are needed to better understand how the attitudes of GPs in Shanghai are shaped by the environments in which they live and work.

**Supplementary Information:**

The online version contains supplementary material available at 10.1186/s12875-022-01716-9.

## Background

Mild cognitive impairment (MCI) has been conceptualised as an intermediate phase between normal cognitive ageing and overt dementia [1]. MCI “*can be divided into amnestic MCI, defined as individuals with a particular impairment of episodic memory often thought to be likely to develop into Alzheimer’s disease, and non-amnestic MCI*” according to the Lancet commission report [2, 3]. MCI converts to dementia at a rate of up to 20% every year [4], with costs of illness more than doubling at the stage of dementia [5]. The American Academy of Neurology recommends that people with MCI be evaluated and monitored in community settings due to their increased risk for developing dementia [6]. In the United Kingdom, it has been estimated that dementia prevalence would be halved if its onset was delayed by five years [2, 7, 8].

There is moderate evidence available on the effectiveness of community-level actions to slow the progress from MCI to dementia, for example, by reducing relevant modifiable risk factors such as insufficient physical activity [9] and depression [10], and properly managing some chronic conditions including diabetes, low serum folate, and neuropsychiatric symptoms [11]. Due to a lack of effective treatment regimens for MCI [12], many community efforts have been focused on non-pharmacological interventions, such as physical exercise [13], Mediterranean diet [14], music intervention [15], and social communication [16]. In addition to these general health improvement measures, there exist three main cognitive intervention approaches [17]: cognitive stimulation therapy [18], cognitive training [19], and cognitive rehabilitation [20]. A recent Cochrane systematic review found that multi-domain interventions may result in some improvement in cognitive function, despite difficulties to rule out potential bias of learning effect. The authors of the review call for further studies into how to implement and sustain multi-domain interventions as “even a very modest effect can have preventive implications when implemented in a population” and these measures have benefits for other health problems such as cardiovascular disease [21].

General practitioners (GPs) are in a unique position to ensure timely detection and effective management of MCI [22]. In many health systems, GPs are designated as the first point of contact for patients to provide cost-effective health services [23]. People with MCI usually present first to their GPs with ambiguous clinical presentations. Following the consensus of a working group comprised of international experts on MCI and Alzheimer’s disease [22], the role of GPs includes identification of MCI, information dissemination about the diagnosis, prognosis, and interventions available for MCI and dementia in the community, as well as referral of patients to specialists for confirmation of MCI diagnosis, and management of behavioural and neuropsychiatric symptoms of MCI patients.

Researchers have developed several theories to explore the underlying mechanisms of medical practice behaviours [24, 25]. The knowledge, attitudes, and practices (KAP) model is a key theory [26]. Other theories are usually built on the KAP model or expand to cover broader environmental factors [27]. The KAP model suggests that any practices are determined by the knowledge and attitudes of the practitioners [26]. Although there is an argument that human behaviours are extremely complex and can be driven by a variety of intrinsic and extrinsic factors [27], it is undeniable that inadequate knowledge and attitudes can harm patients if they are translated into practice. Practitioners’ readiness to practice is critical to prevent unintended detrimental effects when new policies and initiatives are put in place [28].

Several studies have examined the KAP of primary care practitioners in detecting and managing MCI. Empirical evidence shows that even in a system where GPs were well trained, their knowledge of MCI could be limited [29]. Studies found that GPs may acknowledge the value of MCI detection and diagnosis [30, 31], but numerous barriers exist which may result in negative attitudes toward MCI programs among primary care practitioners [32, 33]. Not surprisingly, a significant number of patients with cognitive problems have not been recognised by GPs in their daily practices worldwide [34, 35]. Nevertheless, compared with Canada, the UK, France, Germany, Italy, and Spain, the United States (US) has a higher proportion of primary care physicians who have incorporated cognitive assessment tools into their routine practices [36]. A meta-analysis [37] of eight studies to examine the ability of GPs to recognise MCI in their clinical practices found that about 44.7% of MCI cases could be recognised by GPs. However, only 10.9% of those cases were recorded in medical notes, raising concerns about the lack of attention and actions of GPs on recognised MCI cases.

There is limited understanding of the situation in China, where the clinical role of GPs has been recently developed as the backbone of community health services. This study is one of the few, if any, studies conducted to investigate the KAP of community GPs concerning MCI detection and management in primary care. China launched a nationwide healthcare reform strategy in 2009 [38]. More than 330,000 community clinics and rural township health facilities have been upgraded or reconstructed into community health centres (CHCs) to strengthen its primary care system. The CHCs are supposed to provide primary care services to all residents living within a 15-min transportation radius [39]. The development of CHCs coincides with the process of training (or re-assigning) GPs, who lead the provision of a comprehensive package of primary care services, including the diagnosis and treatment of common diseases, management of chronic conditions, health education, and preventive and rehabilitation care for vulnerable populations. GPs are also assigned a duty to liaise with other care providers and coordinate care on behalf of their patients. Despite a lack of institutional arrangements for the “gatekeeping role” of GPs, CHCs are encouraged to enter into a non-binding health care contract with residents in their designated communities.

Our study was conducted in Shanghai. Shanghai is the first city in China that surpassed the benchmark of an ageing society and experienced negative population growth since 2017 [40]. Shanghai is also taking a leading role in the development of GPs and community health services in China [41]. CHCs in China are charged with responsibilities to serve the rapidly increasing ageing population. In Shanghai, 5.18 million (35.2%) residents were already older than 60 years in 2019 [40]. It is predicted that about 7% of Chinese people over 60 years old (23.3 million) would develop dementia by the year 2030 [42]. The prevalence of MCI in those aged 55 years or older has reached 17.3% and is likely to continue to increase [43]. As one of the earliest cities to establish CHCs, Shanghai is first in line to develop the “Friendly Community Program” for older people with cognitive impairment [44]. In 2018, local governments in Shanghai started to fund public education, risk assessment, early intervention, family support, resource coordination, and information network programs in response to the needs of people with dementia-associated problems.

However, the GP workforce in Shanghai has not been well prepared to respond to the challenge of rapid population aging. Like in other regions of China, GPs are usually employed by CHCs. Prior to the 1990s, many medical practitioners were trained through vocational training programs (equivalent to associate degrees) in China [45]. Some of these practitioners were later on recognised as GPs after completing relevant continuing education training. In 2019, there were 9953 registered GPs employed by 246 CHCs in Shanghai [44]. Although the friendly community programs for the elderly with cognitive impairment are considered highly relevant to community GPs because of their focus on early detection, there have only been a few training workshops on screening and interventions of cognitive disorders that have been open to GPs. Those who attended the training and passed its tests were awarded qualification for MCI screening. However, the absence of the qualification does not make the GPs ineligible for providing MCI screening and intervention services, raising questions about incentives for undertaking the training.

This study aims to:

1. measure the adequacy of knowledge, attitudes, and practice of GPs in Shanghai about MCI detection and management and associations between them;
2. test the mediating role of attitudes in the association between knowledge and practice.
3. identify socio-demographic and work experience factors associated with the KAP of GPs in Shanghai concerning MCI.

## Methods

### Survey instruments

An online questionnaire survey of GPs was conducted in 56 (22.7%) CHCs in Shanghai. The questionnaire was developed through a thorough examination of the existing tools measuring MCI-associated KAP in medical practitioners [30, 46], and adapted to the specific context of China through focus group interviews with 32 MCI patients and 32 caregivers, 42 GPs, and 18 CHC managers in Shanghai. This was followed by two rounds of Delphi consultations with 24 experts with specialisations in general practice, cognitive psychology, dementia care, or health system and services management. The KAP measurements followed the formative assumption rather than the reflective assumption [47], that is, the questionnaire items measure content that is complementary and no item can be removed without compromising the results [48]. The validity of the KAP measurements was assured according to the four criteria recommended by Collier and Bienstock [48]: content specification, indicator specification, indicator collinearity, and external validity. The comprehensive literature review and focus group interviews ensured exhaustive coverage of relevant content. The Delphi consultations enabled clarity about indicator specification and comprehensive coverage of the alternative options of the close-ended questions. The lack of excessive multicollinearity of the indicators was verified (Additional file 1) by the variance inflation factor (VIF) values, which fell far below 10, a commonly used cut-off threshold [49]. In addition, the KAP measurement scales were highly aligned with the existing tools despite adaptation to the Chinese context. For example, the knowledge items covered those included in the “Knowledge of general practitioners on dementia and mild cognitive impairment” [46] and the “dementia knowledge assessment tool” [50]. The attitudes items covered those included in the General Practitioner Attitudes and Confidence Scale [51]. The practice items were categorised as alerting, confirming, and managing domains in line with relevant clinical guidelines used in previous studies [52, 53].

The final version of the questionnaire contained 55 items (Additional file 2). MCI knowledge was tested through 8 question items covering the prevalence, risk factors (4 risks), diagnosis criteria, referral arrangement, screening tool, intervention measures (5 measures), prognosis, and conversion (to dementia) rate of MCI. Respondents were offered opportunities to choose an answer of “unsure” to discourage guessing. Each correct answer was given a score of 1, otherwise, 0 was recorded. A summed score was calculated and transformed into a scoring system ranging from 0 (lowest level of knowledge) to 100 (highest level of knowledge). The respondents were also invited to self-rate their MCI knowledge level on a visual analogue scale, ranging from 0 to 100.

Attitudes were measured with 13 items, tapping into the perceptions of GPs on the nature (susceptibility, severity, benefits of interventions) of the condition, the potential responses from the patient and the society, and the required actions from health care providers. Each item was rated on a five-point Likert scale ranging from 1 “strongly disagree” to 5 “strongly agree”. A summed score was calculated and transformed into a scoring system ranging from 0 (least favourable toward MCI management) to 100 (most favourable toward MCI management).

Compliance with practice guidelines was measured by three domains: alerting, confirming, and managing. The alerting domain contained two questions asking respondents whether they would be alerted to start MCI screening by memory loss and psychiatric symptoms of patients, respectively, for suspected MCI cases. In primary care, these symptoms are dominant patient complaints that lead to the diagnosis of MCI and dementia [46]. Answers to each question included 3 “yes”, 2 “unsure” or 1 “no”. A summed score was calculated for the alerting domain and subsequently transformed into a scoring system ranging from 0 (low alert) to 100 (high alert). The confirming domain contained four items covering family history enquiry, risk factor assessment, MCI screening, and specialist referral for diagnosis. Each item was rated on a five-point Likert scale ranging from 1 “never/not applicable” to 5 “always”. A summed score was calculated for the confirming domain and transformed into a scoring system ranging from 0 (low compliance) to 100 (high compliance). The managing domain contained five questions covering communication with patients and their caregivers, services coordination, medication prescriptions, and non-pharmacological interventions. Each item was rated on a five-point Likert scale ranging from 1 “never/not applicable” to 5 “always”. A summed score was calculated for the managing domain and transformed into a scoring system ranging from 0 (low compliance) to 100 (high compliance). An average score of the three practice domains was eventually calculated, with a higher score indicating higher compliance with relevant clinical guidelines.

Covariates measured in this study included sociodemographic characteristics and the work experience of respondents. Selection of these covariates was informed by previous studies [54]. The sociodemographic characteristics measured were age (< 30, 30–39, 40–49, ≥ 50 years), gender (male, female, others), marital status (married, unmarried, others), educational attainment (below bachelor, bachelor degree, postgraduate), working unit (general practice, public health, health administration, others), years of GP registration (< 5, 5–9, 10–14, ≥ 15), professional title (primary, middle, associate senior, senior), and monthly salary after tax (< 8000, 8000–11,999, 12,000–14,999, ≥ 15,000 Yuan). Work experience measured in this study included volume of daily patient visits (< 80, 80–99, 100–119, ≥ 120), MCI training (yes, no, unsure), MCI screening qualification (yes, no, unsure), the proportion of patients with memory disorder or psychiatric symptoms over the past month (0, < 10%, 10–29%, ≥ 30%, unsure), and past experience in MCI detection and management (yes, no, unsure).

### Study participants and data collection

A stratified cluster sampling strategy was employed to recruit study participants. There are 16 districts in Shanghai, with Pudong as the largest one. The number of CHCs in Pudong is twice the average of other districts. In this study, eight CHCs were randomly selected from Pudong, compared with three or four each from the other districts, in proportion with the district distribution of CHCs.

Data were collected over the period from 13 April to 9 May in 2021. Eligible participants were registered GPs in Shanghai who had direct contact with patients in CHCs. Permission from the senior managers of the identified CHCs was sought through emails before they were asked to invite all of their eligible GPs to participate in the survey. A consent statement explaining the purpose and procedure of the study was attached to the emails for the respondents to read before they decided to proceed with the survey. The survey was conducted via the online platform RedCAP [55]. On average, the survey took around 15 min to complete.

### Ethical considerations

Ethics approval was granted by the Ethics Committee of La Trobe University (HEC20143) in Melbourne and Yangpu Hospital in Shanghai (LL-2019-SCI-004). The study respondents provided implied consent by clicking ‘yes’ to answer the questionnaire. The survey was completely anonymous and respondents were allowed to withdraw at any time and without giving any reason.

### Statistical analysis

The characteristics (categorical variables) of the study participants were described using frequency (%) distributions. Mean (M) and standard deviation (SD) values were calculated to describe MCI-related knowledge, attitudes, and practice of the respondents in addition to frequency (%) distributions of the item responses, and compared among the participants with different characteristics using student *t* or ANOVA tests.

Three logistic regression models were established with knowledge, attitudes, and practice scores (with mean value as a cutoff point) as the dependent variable, respectively. Independent variables entered into the models included the sociodemographic characteristics and work experience of the respondents. Knowledge was included in the regression model for attitudes, while both knowledge and attitudes were included in the regression model for practice. All of the regression models adopted an enter approach. Adjusted odds ratios (AORs) were calculated for potential predictors of higher levels of KAP scores.

The mediating role of attitudes on the association between knowledge and practice was tested using the PROCESS model 4 macro with 5000 bootstrap samples through linear regression modelling [56].

The analyses were performed using IBM SPSS software version 27.0. A two-sided *p* value < 0.05 was considered statistically significant.

## Results

### Sociodemographic characteristics of respondents

A total of 1789 individuals accessed the survey, of whom 49 chose to not participate and 487 withdrew before completing the survey. This resulted in a final sample size of 1253 for data analyses, representing 12.6% of the entire GP workforce in Shanghai [57]. The majority (69.4%) of respondents were women and in the age bracket of 30–49 years (78.8%); only 4.0% did not have a bachelor degree, and 82.8% were married at the time of the survey. The vast majority (93.4%) worked in the department of general practice. About 36.6% had 15 or more years of working experience compared with 25.2% for 10–14 years, 19.7% for 5–9 years, and 18.5% for less than 5 years. Over two-thirds of respondents had a mid-career professional title. Nearly half of respondents had a monthly income of between 8000 and 11,999 Chinese Yuan (Table 1).

**Table 1:** Characteristics of general practitioners (GPs) involved in the study (*n* = 1253)

Variables	N of respondents	% of Respondents	Shanghai GP workforce (2020)
**Age (Years)**			
< 30	149	11.9%	
30–39	541	43.2%	
40–49	446	35.6%	
** ≥ **50	117	9.3%	
**Gender**			
Male	383	30.6%	42.1%
Female	870	69.4%	57.9%
**Marital status**			
Married	1037	82.8%	
Unmarried	193	15.4%	
Others	23	1.8%	
**Educational level**			
< Bachelor	50	4.0%	39.5%
Bachelor degree	990	79.0%	56.2%
Postgraduate degree	213	17.0%	4.3%
**Working unit**			
General practice	1170	93.4%	
Public health	24	1.9%	
Health administration	39	3.1%	
others	20	1.6%	
**Years of GP experience**			
< 5	232	18.5%	
5–9	247	19.7%	
10–14	315	25.2%	
** ≥ **15	459	36.6%	
**Professional title**			
Primary	204	16.3%	46.7%
Middle	829	66.2%	40.4%
Associate senior	196	15.6%	11.2%
Senior	24	1.9%	1.7%
**Monthly income after tax (Yuan)**			
< 8000	481	38.4%	
8000–11,999	558	44.5%	
12,000–14,999	170	13.6%	
** ≥ **15,000	44	3.5%	
**Daily patient visits**			
< 80	820	65.5%	
80–99	297	23.7%	
100–119	107	8.5%	
** ≥ **120	29	2.3%	
**MCI training**			
Yes	367	29.3%	
No	691	55.1%	
Unsure	195	15.6%	
**Qualification of MCI screening**			
Yes	53	4.2%	
No	1116	89.1%	
Unsure	84	6.7%	
**Proportion of patients with memory disorder last month (%)**			
0	111	8.9%	
< 10%	575	45.9%	
10–29%	301	24.0%	
** ≥ **30%	69	5.5%	
Unsure	197	15.7%	
**Proportion of patients with psychiatric symptoms last month (%)**			
0	173	13.8%	
< 10%	717	57.2%	
10–29%	147	11.7%	
** ≥ **30%	26	2.1%	
Unsure	190	15.2%	
**MCI detection and management experience**			
Yes	185	14.8%	
No	915	73.0%	
Unsure	153	12.2%	

Slightly less than 35% of respondents saw more than 80 patients per day on average. Less than 30% received MCI training, but only 4.2% were awarded qualifications for MCI screening. A small percentage of respondents reported no encounters with patients with psychological symptoms (13.8%) or memory complaints (8.9%). However, only 14.8% had been involved in MCI detection and management in the past (Table 1).

### Knowledge, attitudes, and practice of GPs toward MCI detection and management

On average, the respondents obtained an MCI knowledge score of 54.51 (SD = 18.18). Only 0.2% of respondents achieved a full knowledge score, compared with 12.6% obtaining a zero score. Relatively higher levels of knowledge were reflected in the questions associated with risk factors (except for drinking alcohol) and effective intervention measures. However, the understanding of respondents on the prevalence and progression of MCI, MCI screening and diagnosis, and drug therapy was relatively poor, with 13%-40% of respondents providing a correct answer. Less than 30% of respondents understood the criteria for diagnosing MCI (Table 2). The low level of MCI knowledge was also reflected on the self-rated scale: a mean value of 41.23 (SD = 19.98) out of a maximum of 100.

**Table 2:** Knowledge, attitudes, and practice of study participants toward MCI detection and management (*n* = 1253)

**Knowledge item**	**Correct (%)**	**Incorrect (%)**	**Unsure** **(%)**			
MCI prevalence in residents over 60 years	33.4%	66.6%	0			
Risk factors affecting cognitive function (Total)	29.6%	70.4%	0			
Risk 1—lack of exercise	81.1%	18.9%	0			
Risk 2—hearing loss	80.4%	19.6%	0			
Risk 3—depression	89.5%	10.5%	0			
Risk 4—moderate alcohol consumption	33.5%	66.5%	0			
MCI diagnostic criteria	28.6%	47.9%	23.5%			
Referral for MCI diagnosis	37.0%	55.1%	7.9%			
MCI screening tool	34.7%	41.0%	24.3%			
Prognosis of MCI	16.6%	52.4%	31.0%			
MCI conversion rate to dementia	19.5%	43.6%	36.9%			
MCI interventions (Total)	13.7%	86.3%	0			
Aerobic exercise	82.0%	18.0%	0			
Mediterranean diet	55.0%	45.0%	0			
Music	83.6%	16.4%	0			
Social activities	88.3%	11.7%	0			
Effectiveness of Donepezil	40.4%	59.6%	0			
All knowledge items	0.2%	12.6%	0			
Total score (Mean ± SD) of knowledge: 54.51 ± 18.18						
**Attitudes item**	**Strongly agree (%)**	**Agree (%)**	**Neither (%)**	**Disagree (%)**	**Strongly disagree (%)**	
MCI is not a disease, but a degenerative ageing process	2.7%	25.7%	33.5%	32.1%	6.0%	
There are more advantages than disadvantages to finding out if someone has MCI	12.7%	54.8%	26.6%	3.8%	2.1%	
All patients suspected of MCI should undergo a diagnostic evaluation	9.9%	53.2%	31.1%	4.3%	1.5%	
Early recognition and management can delay the progression to Alzheimer’s disease	12.5%	56.3%	27.5%	2.1%	1.6%	
There are more advantages than disadvantages to manage MCI patients with medicine	6.5%	42.6%	42.8%	6.1%	2.0%	
There are more advantages than disadvantages to manage MCI patients with non-pharmaceutical methods	9.7%	53.7%	32.7%	2.4%	1.5%	
Patients with dementia can be a drain on medical and social resources	5.1%	34.1%	44.3%	14.3%	2.2%	
Disclosure of disease could cause stress and frustration to patients and their families	3.3%	35.2%	47.7%	11.7%	2.1%	
Disclosure of disease could cause embarrassment or discomfort for doctors	2.7%	22.6%	42.5%	29.0%	3.2%	
Being diagnosed with MCI could provide some hope for patients compared with being diagnosed with Alzheimer’s disease	3.7%	42.1%	43.9%	8.5%	1.8%	
MCI detection and management provide no economic benefits	6.0%	39.2%	43.2%	9.8%	1.8%	
It’s GPs responsibility to recognise MCI in the primary care setting	5.6%	45.8%	39.1%	6.5%	3.0%	
It’s GPs responsibility to manage MCI in the primary care setting	6.1%	46.0%	38.5%	6.7%	2.7%	
Total score (Mean ± SD) of attitudes: 57.31 ± 7.43						
Practice item						
**MCI alerting practice**	**Yes (%)**	**No (%)**	**Unsure (%)**			
Taking memory disorder as the criteria for MCI detection	42.8%	38.6%	18.6%			
Taking psychiatric symptoms as the criteria for MCI detection	40.4%	35.0%	24.6%			
Total score (Mean ± SD) of alerting practice: 52.39 ± 36.44						
**MCI confirmation practice**	**Always (%)**	**Usually (%)**	**Sometimes (%)**	**Seldom (%)**	**Never** **(%)**	**Not Applicable (%)**
I would ask if a patient has Alzheimer’s` disease family history	18.1%	24.3%	29.3%	20.2%	4.6%	3.5%
I would detect MCI risk factors	12.5%	19.2%	31.2%	24.0%	8.5%	4.6%
I would utilise the MCI screening methods	9.6%	13.7%	25.1%	26.5%	19.1%	6.0%
I would get specialist advice for final diagnosis by transference	15.1%	26.8%	28.2%	18.8%	7.1%	4.0%
Total score (Mean ± SD) of confirming practice: 49.41 ± 26.45						
**MCI management practice**	**Always (%)**	**Usually (%)**	**Sometimes (%)**	**Seldom (%)**	**Never** **(%)**	**Not Applicable (%)**
I would discuss the probable diagnosis with the patient	7.0%	11.9%	28.4%	27.5%	17.3%	7.9%
I would discuss the probable diagnosis with the family	11.1%	20.3%	29.5%	22.8%	10.1%	6.2%
I would coordinate support services	9.6%	17.7%	31.4%	26.7%	9.3%	5.3%
I would prescribe medications	5.1%	8.4%	26.9%	28.3%	22.5%	8.8%
I would provide non-pharmacological interventions	9.5%	17.5%	30.5%	27.0%	9.9%	5.6%
Total score (Mean ± SD) of managing practice: 48.35 ± 17.63						
**Summed average score (Mean ± SD) of practice: 50.05 ± 19.80**						

On average, the respondents had an attitude score of 57.31 (SD = 7.43). The majority of respondents agreed or strongly agreed with MCI management as a strategy to delay the occurrence of Alzheimer’s disease (68.8%) and to support MCI screening (67.5%) and timely diagnosis (63.1%), as well as the adoption of non-pharmaceutical interventions (63.4%). By contrast, a large percentage of respondents (28.4%) would not consider MCI as a disease condition despite 33.5% holding a neutral position. About 45.2% of respondents did not believe that community MCI management would offer any economic benefits. Instead, they were concerned about the potential draining of resources (39.2%) and psychological burdens (38.5%) brought about by MCI screening. Slightly over half respondents agreed and strongly agreed that GPs should take responsibility for detecting and managing MCI in the community (Table 2).

On average, the respondents had a practice score of 50.05 (SD = 19.80) in line with relevant clinical guidelines. Less than half of respondents were likely to be alerted by the presence of memory loss (42.8%) or psychiatric symptoms (40.4%) for suspected MCI cases. Similarly, less than half of respondents would always or usually gather information on family history of Alzheimer’s disease (42.4%) and refer suspected MCI patients to specialists (41.9%). Less than one-third of respondents would always or usually assess risk factors for MCI (31.7%) and perform MCI screening (23.3%). About 19% of respondents would never perform MCI screening. More than 31% of respondents would always or usually discuss MCI diagnosis with family members, compared with 18.9% with patients themselves. About half of the respondents would take non-pharmacological measures (Table 2).

### Factors associated with knowledge, attitudes, and practice

Age was associated with attitudes (*p* = 0.001) and practice (*p* = 0.013) scores, but not in a linear manner. Higher knowledge and attitude scores were found in the female participants (*p* < 0.05). Higher knowledge scores were also found in those who were not married (*p* = 0.028), had a university degree (*p* = 0.005), and earned a higher income (*p* = 0.008) (Table 3).

**Table 3:** MCI knowledge, attitude, and practice scores by sociodemographic characteristics of study participants

Characteristics	Knowledge scores				Attitude scores				Practice scores			
	**Mean**	**SD**	***F/t***	***p***	**Mean**	**SD**	***F/t***	***p***	**Mean**	**SD**	***F/t***	***p***
Age (Years)												
< 30	54.55	18.063	2.098	0.099	58.19	8.107	5.270	**0.001**	54.23	21.786	-3.606	**0.013**
30–39	55.24	18.706			56.41	7.123			48.42	19.495		
40–49	54.63	17.710			58.15	7.312			50.66	19.404		
≥ 50	50.61	17.359			57.14	7.985			49.94	19.344		
Gender												
Male	52.83	19.598	2.166	**0.030**	56.22	7.452	3.465	**0.001**	49.67	19.033	0.455	0.649
Female	55.25	17.482			57.79	7.378			50.22	20.131		
Marriage												
Married	54.00	18.189	2.197	**0.028**	57.24	7.381	0.678	0.498	49.57	19.453	1.899	0.058
Unmarried	56.98	17.984			57.62	7.689			52.37	21.260		
Education												
Under bachelor	48.00	19.363	5.260	**0.005**	58.48	8.791	-1.186	0.306	52.30	20.719	-0.581	0.559
Bachelor	54.31	17.825			57.36	7.220			49.77	19.695		
Postgraduates	52.83	19.598			56.22	7.452			49.67	19.033		
Department												
General practice	54.32	18.339	1.411	0.159	57.17	7.309	2.449	**0.014**	49.96	19.729	0.631	0.528
Others	57.23	15.637			59.24	8.827			51.38	20.798		
Experience (Years)												
< 5	55.02	18.719	1.469	0.221	58.13	7.736	-2.886	**0.035**	51.58	21.298	-1.863	1.340
5–9	54.31	19.263			56.69	7.522			47.54	19.187		
10–14	56.03	18.364			56.61	7.090			50.34	19.599		
≥ 15	53.31	17.124			57.71	7.413			50.43	19.406		
Professional title												
primary	48.00	19.363	1.926	0.124	58.48	8.791	-5.497	**0.001**	52.30	20.719	-3.932	**0.008**
Middle	54.31	17.825			57.36	7.220			49.77	19.695		
Senior	56.98	19.157			56.78	8.040			50.82	20.083		
Income (Yuan)												
< 8000	52.82	19.219	3.938	**0.008**	56.33	7.416	6.423	**0.001**	50.56	20.397	2.359	0.070
8000–11,999	54.76	17.715			57.62	7.141			48.61	19.390		
≥ 12,000	56.97	16.018			58.37	7.842			52.81	19.191		
Daily visits												
< 80	54.18	18.179	0.847	0.468	57.27	7.581	0.184	0.907	50.06	20.529	0.079	0.971
80–99	54.52	17.373			57.26	6.649			50.01	18.154		
≥ 100	56.46	19.861			57.65	8.167			50.08	18.839		
MCI Training												
With	59.58	15.594	6.451	< **0.001**	57.94	8.105	1.925	0.054	54.79	19.267	5.515	< **0.001**
Without	52.41	19.762			57.05	7.125			48.09	19.690		
Qualification of MCI detection												
With	63.61	19.525	3.744	< **0.001**	57.23	9.402	-0.077	0.938	61.21	19.526	4.222	< **0.001**
Without	54.11	18.023			57.31	7.339			49.56	19.670		
Proportion of patients with memory disorder last month												
Unsure	43.58	22.599	20.638	< **0.001**	54.90	6.149	5.359	< **0.001**	43.25	17.887	6.738	< **0.001**
zero	52.64	19.043			59.06	7.481			50.16	22.783		
< 10%	56.12	15.703			57.57	6.696			50.87	19.938		
10–29%	59.02	15.791			57.94	8.605			51.42	18.430		
≥ 30%	55.59	19.518			58.11	9.586			56.46	20.236		
Proportion of patients with psychiatric symptoms last month												
Unsure	42.97	21.766	21.995	< **0.001**	54.81	6.269	6.409	< **0.001**	43.62	19.312	6.125	< **0.001**
zero	55.37	19.197			57.19	7.627			49.88	22.843		
< 10%	56.84	16.063			58.08	7.480			50.88	19.486		
10–29%	58.21	16.581			57.21	7.706			53.25	17.411		
≥ 30%	47.80	19.976			55.56	7.239			57.36	20.130		
MCI detection and management experience												
With	58.22	17.715	3.020	**0.003**	58.97	8.248	3.301	**0.001**	62.38	18.333	9.498	< **0.001**
Without/unsure	53.87	18.192			57.02	7.248			49.91	19.260		
Knowledge scores												
High					58.50	7.582	6.958	< **0.001**	52.83	18.815	6.076	< **0.001**
Low					55.58	6.861			46.02	20.493		
Attitude scores												
High	58.17	15.932	7.867	< **0.001**					53.74	19.662	7.254	< **0.001**
Low	50.26	19.660							45.77	19.092		

The participants who had experience in MCI management had higher KAP scores (*p* < 0.01). Those who attended MCI training (*p* < 0.001) and had a qualification for MCI screening (*p* < 0.001) had higher knowledge and practice scores, despite a lack of difference in attitude scores (*p* = 0.938). The participants working in the department of general practice (*p* = 0.014) and those who had 5–14 years of working experience (*p* = 0.035) and earned a lower income (*p* = 0.001) had lower attitude scores than others. The primary professional title was associated with higher attitude and practice scores (*p* < 0.01). The KAP scores of the GPs varied by the proportion of their patients with memory disorder or psychiatric symptoms over the past month (*p* < 0.001), but there is not a linear correlation (Table 3).

The multivariate logistic regression models confirmed that MCI training (AOR = 1.553, *p* = 0.003) and senior professional title (AOR = 1.850, *p* = 0.043) were associated with higher levels of knowledge. Unsure about the proportions of patients who had memory disorders and psychiatric symptoms was associated with lower levels of MCI knowledge (*p* < 0.05).

Favourable attitudes toward MCI detection and management were associated with higher levels of MCI knowledge (AOR = 1.974, *p* < 0.001). Female GPs (AOR = 1.551, *p* = 0.001) and those who earned more than 8000 Yuan (AOR = 1.557–2.251, *p* < 0.01) held more favourable attitudes toward MCI detection and management. Past experience in MCI detection and management was also associated with more favourable attitudes (AOR = 1.582, *p* = 0.014), although MCI screening qualification was associated with less favourable attitudes (AOR = 0.452, *p* = 0.014).

Higher levels of compliance in practice were found in the respondents with higher knowledge (AOR = 1.426, *p* = 0.006) and more favourable attitudes (AOR = 2.095, *p* < 0.001). Past experience (AOR = 3.034, *p* < 0.001) and MCI screening qualification (AOR = 2.162, *p* = 0.035) were both significant predictors of higher compliance in practice. Unsure about the proportion of patients who had memory disorders was associated with lower compliance in practice (*p* < 0.05) (Table 4).

**Table 4:** Logistic regression results of predictors of MCI knowledge, attitudes and practice scores

	**Knowledge level** **(High vs Low)**				**Attitude level** **(More vs Less Favourable)**				**Practice level** **(High vs Low Compliance)**			
	**Unadjusted OR**	**AOR**	**95%CI**	***p***	**Unadjusted OR**	**AOR**	**95%CI**	***p***	**Unadjusted OR**	**AOR**	**95%CI**	***p***
**Age (Years)**												
< 30	1	1			1	1			1	1		
30–39	1.178	1.093	0.615–1.943	0.761	0.637	0.760	0.431–1.340	0.343	0.578	0.568	0.321–1.004	0.052
40–49	1.114	1.036	0.530–2.022	0.918	1.015	1.148	0.592–2.230	0.683	0.733	0.723	0.372–1.408	0.341
≥ 50	0.715	0.701	0.321–1.531	0.373	0.755	0.902	0.414–1.966	0.795	0.740	0.807	0.368–1.771	0.593
**Gender**												
Male	1	1			1	1			1	1		
Female	1.159	1.186	0.916–1.535	0.195	1.443	1.551	1.196–2.012	**0.001**	1.043	1.005	0.771–1.309	0.971
**Marriage**												
Unmarried	1				1	1			1	1		
Married	0.785	1.427	0.989–2.057	0.057	0.854	0.918	0.641–1.315	0.640	0.819	0.898	0.629–1.288	0.559
**Education**												
Under bachelor	1	1			1	1			1	1		
Bachelor	1.654	1.231	0.652–2.324	0.521	1.103	0.861	0.451–1.642	0.649	0.821	1.195	0.620–2.300	0.595
postgraduates	2.205	1.411	0.693–2.873	0.343	0.949	0.636	0.309–1.308	0.219	0.914	1.350	0.649–2.810	0.422
**Department**												
Others	1	1			1	1			1	1		
General practice	0.854	1.277	0.764–2.134	0.351	1.083	0.972	0.583–1.620	0.912	0.980	1.062	0.638–1.769	0.817
**Experience (years)**												
< 5	1	1			1	1			1	1		
5–9	0.785	0.670	0.431–1.040	0.074	0.678	0.741	0.480–1.144	0.176	0.603	0.832	0.534–1.296	0.416
10–14	0.962	0.771	0.478–1.242	0.285	0.675	0.646	0.404–1.033	0.068	0.841	1.244	0.773–2.004	0.369
≥ 15	0.798	0.648	0.378–1.110	0.114	0.939	0.609	0.357–1.042	0.070	0.867	1.112	0.646–1.912	0.702
**Professional title**												
Primary	1	1			1	1			1	1		
Middle	1.158	1.388	0.852–2.261	0.187	0.765	0.882	0.544–1.428	0.608	0.752	0.895	0.549–1.460	0.657
Senior	1.556	1.850	1.020–3.356	**0.043**	1.527	1.399	0.774–2.527	0.266	0.889	0.650	0.357–1.184	0.159
**Income (Yuan)**												
< 8000	1	1			1	1			1	1		
8000–11,999	1.119	1.068	0.811–1.407	0.639	1.359	1.557	1.182–2.050	**0.002**	0.906	0.894	0.675–1.185	0.436
≥ 12,000	1.328	1.176	0.801–1.725	0.408	2.117	2.251	1.526–3.321	** < 0.001**	1.327	1.213	0.824–1.787	0.327
**Daily visits**												
< 80	1	1			1	1			1	1		
80–99	1.166	1.131	0.838–1.526	0.422	0.946	0.889	0.660–1.197	0.438	0.884	0.822	0.607–1.114	0.207
≥ 100	1.137	1.000	0.669–1.494	0.999	1.051	0.952	0.639–1.419	0.809	0.971	0.763	0.505–1.151	0.197
**MCI training**												
No and unsure	1	1			1	1			1	1		
Yes	1.779	1.553	1.162–2.075	**0.003**	1.184	1.023	0.768–1.361	0.877	1.664	1.203	0.903–1.603	0.207
**MCI detection and treatment experience**												
No and unsure	1	1			1	1			1	1		
Yes	1.445	1.042	0.723–1.503	0.824	1.584	1.582	1.096–2.285	**0.014**	3.758	3.034	2.068–4.453	** < 0.001**
**MCI screening qualification**												
No and unsure	1	1			1	1			1	1		
Yes	1.791	1.117	0.567–2.201	0.749	0.703	0.452	0.240–0.852	**0.014**	3.540	2.162	1.055–4.431	**0.035**
**Memory loss encounter**												
Unsure	1	1			1	1			1	1		
Zero	1.757	0.865	0.436–1.717	0.678	2.506	1.501	0.744–3.027	0.256	1.872	1.420	0.694–2.905	0.337
< 10%	2.290	1.298	0.787–2.140	0.307	2.026	1.121	0.675–1.861	0.660	2.260	1.620	0.954–2.751	0.074
10–29%	3.558	1.936	1.143–3.280	**0.014**	1.686	0.948	0.558–1.611	0.845	2.489	1.761	1.015–3.054	**0.044**
≥ 30%	2.323	1.525	0.765–3.039	0.230	2.104	1.591	0.791–3.199	0.192	4.281	2.857	1.396–5.849	**0.004**
**Psychiatric symptom encounter**												
Unsure	1	1			1	1			1	1		
Zero	0.610	2.213	1.183–4.141	**0.013**	2.466	1.609	0.852–3.038	0.142	1.779	0.898	0.467–1.728	0.748
< 10%	1.471	2.024	1.224–3.348	**0.006**	2.290	1.747	1.046–2.918	0.033	2.247	1.080	0.635–1.838	0.775
10–29%	1.737	2.065	1.135–3.758	**0.018**	1.699	1.364	0.748–2.485	0.311	2.449	1.081	0.583–2.004	0.806
≥ 30%	2.000	1.218	0.476–3.120	0.681	1.229	1.003	0.387–2.602	0.995	4.093	1.995	0.737–5.400	0.174
**Knowledge score levels**												
Low					1	1			1	1		
High					2.063	1.974	1.543–2.526	** < 0.001**	1.762	1.426	1.106–1.837	**0.006**
**Attitudes score levels**												
Low									1	1		
High									2.303	2.095	1.631–2.690	** < 0.001**
**Adjusted R-squared**		0.075				0.101				0.129		

The linear regression models showed that attitudes partially mediated the association between knowledge and practice (*p* < 0.001) after adjustment for variations in other independent variables, with 19.7% of the total effect being explained by the indirect effect (Additional file 3).

## Discussion

Findings of this study indicate that GPs in Shanghai have a low level of MCI knowledge, do not hold clearly favourable attitudes toward community detection and management of MCI, and have self-reported low compliance with relevant clinical guidelines in practice. None of the KAP scores exceed 60 out of a maximum of 100. It is important to note that male GPs (30.6% vs 42.1% in Shanghai) and those who held a primary professional title (16.3% vs 46.7% in Shanghai) and did not have a bachelor degree (4% vs 29.5% in Shanghai) were under-represented in the study sample [57]. This could lead to an overestimation of KAP scores. The actual knowledge, attitudes, and practice in the GP workforce in Shanghai are likely to be more concerning than results revealed in this study.

Poor MCI knowledge of GPs is a worldwide concern. In our study, only 0.2% of respondents achieved a full knowledge score while 12.6% obtained a zero score. Less than 30% understood the criteria for diagnosing MCI. In contrast with quiz tests of GPs’ knowledge of dementia in England and Greece [58, 59], the overall level of MCI knowledge was found to be low among GPs in our study. A study conducted in Israel revealed 197 GPs held a moderate level of (mean = 3.5, SD = 1.7, range = 0–8) MCI knowledge [29]. In addition to poor test results, GPs in Italy felt that they were not well-informed and trained [60]. Similar to the findings of MCI studies in German and Israel [29, 35], we found that GPs tend to underestimate the prevalence of MCI.

Knowledge can be accumulated through learning and work experience. Indeed, targeted training was found to be associated with higher MCI knowledge scores in this study. The effectiveness of training on MCI knowledge gain has been demonstrated in a systematic review [61]. In our study, less than 30% of respondents reported having received MCI training. The percentage of GPs being awarded a qualification for MCI screening on completion of training is even lower at a level of 5%. Moreover, holding such a qualification is not a significant and/or reliable predictor of MCI knowledge. In our study, less than 15% of respondents reported past experience in detecting and managing MCI, and a similarly low level of experience detecting MCI was found in Australia and New Zealand [62], Israel [29], and some other European countries [35]. Although past experience is not a significant predictor of MCI knowledge, we found that unsure about the proportions of their patients with memory complaints and psychiatric symptoms among the GPs is associated with lower levels of knowledge. This may be a proxy indication of lower awareness of suspected cases of MCI. A study in Hungary [46] showed that most GPs identified memory complaints (63.1%) as a key trigger leading to the diagnosis of MCI.

Overall, according to the findings of this study, GPs in Shanghai did not show clearly favourable attitudes toward MCI detection and management. The average attitudes score (57.31 ± 7.43) is relatively low. Although only one third of our study respondents did not consider MCI as a disease, lower than those reported in Israel (70%) [29] and in Brazil (92%) [30], over one third (33%) of our study respondents held a neutral position. Previous studies showed that the perception of patients (and family members) that MCI is part of normal aging accounted for over half of GPs’ hesitancy to detect and diagnose MCI [33]. There is a positive sign though that over half of our study respondents endorsed their role in MCI detection and management, compared with less than 10% clearly opposing this role. However, there exist significant barriers for GPs to fulfill this role. We found in this study that a large proportion of GPs worried about disclosure of MCI diagnosis to patients and families (38.5%) and were skeptical about the economic benefits of MCI detection (45.2%), in contradiction to the evidence that MCI detection and management is cost-effective [63].

We found that lower levels of MCI knowledge are associated with unfavorable attitudes. This result is consistent with the findings of previous studies [53]. In theory, underestimating the prevalence and seriousness of MCI may jeopardise recognition of the importance of and endorsement from GPs for the community detection and management of MCI. A lack of knowledge and confidence concerning MCI detection and management may also disempower GPs and result in a pessimistic mindset. Doubting the validity of diagnostic criteria for MCI, and realising there is uncertainty about the progression of MCI into dementia, may exacerbate GPs’ hesitancy about performing MCI screening and diagnosis. Harmand and colleagues found that the limited effectiveness of drug therapy is a major reason contributing to underdiagnosis of MCI in primary care in France [64]. Some previous studies found that more highly qualified and more experienced GPs are more likely to support MCI detection and management [65]. However, our study shows that higher income and longer working experience are associated with more favourable attitudes toward MCI detection and management, but MCI screening qualification is associated with unfavourable attitudes. The underlying reason for this finding is not clear. We suspect that those with an MCI screening qualification may be more aware of the extrinsic barriers.

Low compliance with practice guidelines is evident in the self-reported practice intentions of GPs concerning community detection and management of MCI. This finding is similar to those found in a cross-sectional study in Germany [35], in which GPs could recognise MCI in a very limited number of cases based on clinical impression only. Around 40% of our study respondents would take memory loss or psychiatric symptoms as a trigger for MCI detection, compared with 63.1% of 402 GPs in Hungary who marked memory complaints as MCI symptoms [46]. A person can only become part of a community MCI management program when they receive confirmation of an MCI diagnosis following the screening. Unfortunately, our study found that less than one-third of study respondents would perform MCI screening, while 19.1% would never perform MCI screening. In addition, there exists some confusion among the GPs regarding where to refer suspected patients for diagnostic confirmation: 37.0% answered the item correctly. These results are consistent with the findings of another study conducted in five European countries, Canada, and the US [36]. A survey conducted in Australia and New Zealand showed that clinicians believe that the perception of a lack of effective treatments for MCI is a serious barrier to encouraging consumers to accept MCI screening [62]. There is also a dilemma associated with patient communication. Consistent with the findings of a study conducted in German [35], GPs prefer to disclose MCI diagnosis to their caregivers rather than the patients themselves.

Findings of this study support the proposed KAP model. Both knowledge and attitudes are associated with the practice, while attitudes partially mediate the association between knowledge and practice. A lack of proper knowledge has been listed as a major contributing factor to the underdiagnosis of MCI worldwide [29, 36, 46]. Incorrect answers given by more than 40% of our study respondents regarding MCI screening and diagnostic criteria are particularly concerning. This is likely to fuel hesitancy or lead to diagnostic errors. It is indeed a concern that only 67.8% GPs in this study reported endorsement of the effectiveness of MCI screening in delaying the occurrence of dementia.

There are some policy and practice implications from this study. The low level of knowledge, lack of favourable attitudes, and low compliance with practice guidelines together present a serious challenge to the implementation of MCI-related initiatives in Shanghai. Internationally, training has often been adopted as a major strategy for improving professional knowledge and encouraging best practice. Although we found that training is a significant predictor of knowledge, it failed to predict attitudes and practice. In addition, it is important to note that the independent variables, including knowledge and attitudes, could only explain less than 20% of variations in the practice score. Clearly, training alone is not enough. This points to the limitations of the KAP model, despite its popularity. Empirical evidence shows that human behaviours are not always aligned with individuals’ knowledge [66]. Health workers often succumb to the strong influence of professional culture and subjective norms [24]. Their choices of actions are also shaped by regulations, policies, rules, and pressures from consumers [67]. In China, financial incentives are often used for motivating health professionals to engage in best practices [68]. In this study, however, we did not find income to be a significant predictor of practice although higher income is associated with more favorable attitudes toward MCI detection and management. Contemporary theories in human resources management often encourage a comprehensive set of measures that enhance purpose, autonomy, and mastery [69]. Actions such as training, management support, team efforts, and financial incentives are supposed to reinforce each other to achieve optimal outcomes. Meanwhile, such measures need to be tailored to the varying needs of health workers, as illustrated by the findings of this study and others [58]. According to the findings of this study and others [70], male and junior GPs appear to need more attention as they have lower MCI knowledge or less favourable attitudes. Empirical evidence suggests that MCI training alone can be perceived as inadequate [71] and it will become effective only when it is linked with organisational incentives and supportive environments [72].

### Strengths and limitations

This study is the first of its kind to assess the knowledge, attitudes, and practice of GPs in Shanghai regarding community detection and management of MCI. Even though it follows a stringent protocol in study design and tool development, several limitations are acknowledged so that the findings of this study can be adequately interpreted. Firstly, data were collected using a self-report questionnaire, which may produce some recall bias and reporting errors. Voluntary participation and anonymity were adopted as a strategy in this study to mitigate the risk of reporting errors. Secondly, the study adopted a cross-sectional design, which prevented us from drawing causal conclusions. Thirdly, there was an over-representation of female GPs and over-representation of GPs who had a university medical degree in this study sample compared with the GP workforce in Shanghai. This may result in an overestimation of the knowledge level of GPs, although the overall knowledge level of this study sample concerning MCI is already deemed low. Finally, Shanghai is one of the most developed regions in China. Attempts to generalise the findings to other parts of China need to be done cautiously.

## Conclusion

MCI detection and management in primary care is deemed crucial for the control and mitigation of health consequences resulting from dementia. Under the circumstance of an ageing society in Shanghai and a higher prevalence of MCI, our study shows that GPs in Shanghai are not well prepared to undertake such a role. Overall, their MCI knowledge level is low. There is a lack of clearly favourable attitudes toward MCI detection and management. The reported practice intentions of the GPs indicate low compliance with practice guidelines. Although MCI training can and should play an important role in the development of community MCI management programs, training by itself is not enough. Training alone does not foster positive attitudes that mediate the association between knowledge and practice. Future studies should consider a more complex model, such as the Chronic Care Model [72], which applies a system thinking approach by taking the broader environmental context into account. Management of chronic conditions, such as MCI, requires a systems approach involving the patients, their care providers, and a platform that enables effective interactions between the two. This calls for multidisciplinary professional collaborations and partnership between patients and care providers. Although the major purpose of such a team-based partnership approach is to improve the quality (e.g. continuity and coordination) of care, it may also bring benefits to the patients in resource-poor settings through its “offset effect” on the demand of the already scarce infrastructure and qualified medical workforce [73]. Further studies are needed to better understand how GPs perceive and respond to the needs of various “team members”.

## Supplementary Information

**Additional file 1: Appendix File 1.** The variance inflation factor (VIF) values for the Multicollinearity analyses.

**Additional file 2:** **Appendix File 2.** Survey on the detection and management of mild cognitive impairment in community health services.

**Additional file 3: Appendix File 3.** Mediation regression results of predictors of MCI knowledge on practice scores via attitudes.

## Data Availability

The datasets generated and analysed during the current study are available from the corresponding author CL on reasonable request.

## Abbreviations

GPs: General practitioners
MCI: Mild cognitive impairment
KAP: Knowledge, attitudes and practice
CHCs: Community health centres
CCM: The Chronic Care Model
