# Supplementary material for: Knowledge, attitudes, and practice of general practitioners toward community detection and management of mild cognitive impairment: a cross-sectional study in Shanghai, China
Source: BMC Prim Care. 2022 May 11;23:114. doi: 10.1186/s12875-022-01716-9 (PMC9092880; doi:10.1186/s12875-022-01716-9)
Supplement: Supplementary file 1 — Additional file 1: Appendix File 1. The variance inflation factor (VIF) values for the Multicollinearity analyses. [file 12875_2022_1716_MOESM1_ESM.docx]

**Appendix File 1. The variance inflation factor (VIF) values for the Multicollinearity analyses**

| **Construct Name** | **Code** | **Mean** | **SD** | **Tolerance** | **VIF** |
| --- | --- | --- | --- | --- | --- |
| **Knowledge**  **(Condition Index=4.280)** | MCI prevalence | 0.531 | 0.058 | 0.935 | 1.069 |
|  | Risk factors | 0.284 | 0.08 | 0.949 | 1.054 |
|  | MCI diagnosis | 0.424 | 0.075 | 0.971 | 1.030 |
|  | MCI Referral | 0.289 | 0.074 | 0.985 | 1.015 |
|  | Screening tool | 0.552 | 0.054 | 0.934 | 1.071 |
|  | Prognosis | 0.311 | 0.082 | 0.957 | 1.045 |
|  | Conversion rate | 0.621 | 0.053 | 0.921 | 1.086 |
|  | MCI Intervention | 0.276 | 0.069 | 0.961 | 1.041 |
| **Attitudes**  **(Condition Index=13.282)** | Normal Aging | 0.099 | 0.100 | 0.798 | 1.253 |
|  | Screen Benefit | 0.812 | 0.061 | 0.324 | 3.091 |
|  | Confirmation Needed | 0.817 | 0.061 | 0.302 | 3.314 |
|  | Dementia Defer Possible | 0.865 | 0.062 | 0.229 | 4.360 |
|  | Pharmacological Benefit | -0.563 | 0.082 | 0.575 | 1.738 |
|  | Non-pharmacological Benefit | 0.785 | 0.069 | 0.373 | 2.681 |
|  | Waste Resources | -0.186 | 0.124 | 0.528 | 1.893 |
|  | Patient Disturbance | -0.263 | 0.127 | 0.478 | 2.092 |
|  | GP Awkward | 0.054 | 0.135 | 0.511 | 1.956 |
|  | Patient Hope | 0.480 | 0.099 | 0.551 | 1.815 |
|  | Lack Financial Benefit | -0.290 | 0.113 | 0.613 | 1.631 |
|  | Screening Obligation | 0.723 | 0.059 | 0.200 | 5.004 |
|  | Intervention Obligation | 0.742 | 0.057 | 0.198 | 5.039 |
| **Practice**  **(Condition Index=10.941)** | Family History | 0.835 | 0.012 | 0.333 | 3.003 |
|  | Risk Factors | 0.887 | 0.008 | 0.224 | 4.464 |
|  | Screening | 0.825 | 0.017 | 0.280 | 3.566 |
|  | Informing Patients | 0.727 | 0.026 | 0.406 | 2.462 |
|  | Informing Caregivers | 0.856 | 0.014 | 0.285 | 3.509 |
|  | Referral | 0.785 | 0.018 | 0.418 | 2.392 |
|  | Coordinate | 0.883 | 0.011 | 0.214 | 4.677 |
|  | Prescribe | -0.659 | 0.029 | 0.467 | 2.143 |
|  | Non-pharmacological Intervention | 0.854 | 0.012 | 0.273 | 3.668 |
|  | Alert Memory Loss | 0.205 | 0.043 | 0.861 | 1.161 |
|  | Alert Psychological Symptom | 0.238 | 0.043 | 0.849 | 1.178 |
