# Supplementary material for: Knowledge, attitudes, and practice of general practitioners toward community detection and management of mild cognitive impairment: a cross-sectional study in Shanghai, China
Source: BMC Prim Care. 2022 May 11;23:114. doi: 10.1186/s12875-022-01716-9 (PMC9092880; doi:10.1186/s12875-022-01716-9)
Supplement: Supplementary file 2 — Additional file 2: Appendix File 2. Survey on the detection and management of mild cognitive impairment in community health services. [file 12875_2022_1716_MOESM2_ESM.doc]

Survey on the detection and management of mild cognitive impairment in community health services

Dear physicians:

Greeting! We are conducting a questionnaire survey on the preparedness of general practice in detecting and managing mild cognitive impairment (MCI) in Shanghai. You are invited to complete this 20-minute questionnaire. Your responses are of great values. We hope to learn your experience and perceptions, which will help us determine how general practitioners (GPs) can effectively detect and manage MCI in Shanghai. The survey is completely voluntary and anonymously. Your responses will only be used for academic research only.

If you are interested in the survey, please read the following information statement carefully. Thank you very much for your participation.

Please make sure whether you`d like to take this survey：

**☐ Yes**

**☐ No**

**Participant characteristics**

1. **Gender**

☐Male

☐Female

☐Others

1. **Age**

☐ < 30
☐ 30-39
☐ 40-49
☐ ≥ 50

1. **Marital status**

☐Married

☐Unmarried

☐Other

1. **Educational level**

☐ Under bachelor

☐ Bachelor

☐ Postgraduate

1. **Working department**

☐General practice

☐ Public health

☐ Health administration

☐ Others

1. **Length of GP experience**

☐ < 5
☐ 5-9
☐ 10-14
☐ ≥15

1. **Professional title**

☐ Primary

☐ Middle

☐ Associate senior

☐ Senior

1. **Monthly income after tax**

☐ < ¥8000
☐ ¥8000-11999
☐ ¥12000-14999

☐ ≥ ¥15000

**MCI detection and management context experience**

1. **Daily visiting patients**

☐ < 80

☐ 80-99

☐ 100-119

☐ ≥120

1. **MCI training experience**

☐ Yes

☐ No

☐ Unsure

1. **MCI detection qualification**

☐Yes

☐No

☐Unsure

1. **Proportion of patients with memory disorder last month**

☐ 0

☐ <10%

☐ 10-29%

☐ ≥ 30%

☐ Unsure

1. **Proportion of patients with psychiatric symptoms last month**

☐ 0

☐ <10%

☐ 10-29%

☐ ≥ 30%

☐ Unsure

1. **MCI detection and management experience**

☐ Yes

☐ No

☐ Unsure

**Knowledge of GPs toward MCI detection and management**

1. **What is the estimated percentage of people over 60 years old who have MCI in community setting?**

☐ <10%

☐ 10-29%

☐ ≥ 30%

☐ Unsure

1. **Which are modifiable risk factors affecting cognitive function?**

☐ lack of exercise

☐ hearing loss

☐ depression

☐ moderate drink

☐ Unsure

1. **Which of the following is** **NOT required to diagnose MCI according to Peterson diagnosis criteria?**

☐ Subjective memory loss

☐ Impaired activity of daily living

☐ Evidence of cognitive deterioration

☐ Not clinically demented

☐ Unsure

1. **Which of the following doctors are necessary in MCI diagnosis?**

☐ Psychologist

☐ Psychiatrist

☐ Neurologist

☐ All of the above

☐ Unsure

1. **Which one of the following is the most commonly-used MCI screening scale?**

☐ The Montreal Cognitive Assessment (MoCA)

☐ Mini-Mental State Examination (MMSE)

☐ Clinic Dementia Rating Score (CDR)

☐ Activities of Daily Living (ADL)

☐ Unsure

1. **Which statement is false concerning treatment of MCI?**

☐ Donepezil should be prescribed

☐ Management of MCI may reverse symptoms

☐ It is important to rule out and treat reversible risks

☐ The cognitive function may reverse automatically in some MCI cases

☐ Unsure

1. **What is the average progression rate after onset of MCI?**

☐ <10%

☐ 10-19%

☐ 20-39%

☐ ≥40%

☐ Unsure

1. **What are effective non-pharmacological MCI interventions?**

☐ Aerobic exercise

☐ Mediterranean diet

☐ Music

☐ Social activities

☐ Donepezil effective effect

**Attitudes of GPs toward MCI detection and management**

|  | **Strongly disagree** | **Disagree** | **Unsure** | **Agree** | **Strongly Agree** |
| --- | --- | --- | --- | --- | --- |
| 1. MCI is not a disease, but a degenerative aging process. | ☐ | ☐ | ☐ | ☐ | ☐ |
| 2. There are more advantages than disadvantages to finding out if someone has MCI. | ☐ | ☐ | ☐ | ☐ | ☐ |
| 3. All patients suspected of MCI should undergo a diagnostic evaluation. | ☐ | ☐ | ☐ | ☐ | ☐ |
| 4. Early recognition and management can delay the progression to Alzheimer`s disease. | ☐ | ☐ | ☐ | ☐ | ☐ |
| 5. There are more advantages than disadvantages to manage MCI patients with medicine. | ☐ | ☐ | ☐ | ☐ | ☐ |
| 6. There are more advantages than disadvantages to manage MCI patients with non-pharmaceutical methods. | ☐ | ☐ | ☐ | ☐ | ☐ |
| 7. Patients with dementia can be a drain on medical and social resources | ☐ | ☐ | ☐ | ☐ | ☐ |
| 8. Disclosure of disease could cause stress and frustration to patients and their families. | ☐ | ☐ | ☐ | ☐ | ☐ |
| 9. Disclosure of disease could cause embarrassment or discomfort for doctors. | ☐ | ☐ | ☐ | ☐ | ☐ |
| 10. Being diagnosed with MCI could provide some hope for patients compared with being diagnosed with Alzheimer’s` disease. | ☐ | ☐ | ☐ | ☐ | ☐ |
| 11. MCI detection and management provide no economic benefits. | ☐ | ☐ | ☐ | ☐ | ☐ |
| 12. It`s GPs responsibility to recognise MCI in the primary care setting. | ☐ | ☐ | ☐ | ☐ | ☐ |
| 13. It`s GPs responsibility to managing MCI in the primary care setting. | ☐ | ☐ | ☐ | ☐ | ☐ |

Practice of GPs toward MCI detection and management

|  | **Always** | **Usually’** | **Sometimes** | **Seldom** | **Never** | **Not Applicable** |
| --- | --- | --- | --- | --- | --- | --- |
| 1. I take memory disorder as the criteria for MCI detection | ☐ | ☐ | ☐ | ☐ | ☐ | ☐ |
| 2. I take psychiatric symptoms as the criteria for MCI detection | ☐ | ☐ | ☐ | ☐ | ☐ | ☐ |
| 3. I would ask if a patient has Alzheimer’s` disease family history | ☐ | ☐ | ☐ | ☐ | ☐ | ☐ |
| 4. I would detect MCI risk factors | ☐ | ☐ | ☐ | ☐ | ☐ | ☐ |
| 5. I would utilise the MCI screening methods | ☐ | ☐ | ☐ | ☐ | ☐ | ☐ |
| 6. I would get specialist advice for final diagnosis by transference | ☐ | ☐ | ☐ | ☐ | ☐ | ☐ |
| 7. I would discuss the probable diagnosis with the patient. | ☐ | ☐ | ☐ | ☐ | ☐ | ☐ |
| 8. I would discuss the probable diagnosis with the family. | ☐ | ☐ | ☐ | ☐ | ☐ | ☐ |
| 9. I would coordinate support services. | ☐ | ☐ | ☐ | ☐ | ☐ | ☐ |
| 10. I would prescribe medications | ☐ | ☐ | ☐ | ☐ | ☐ | ☐ |
| 11. I would provide non-pharmacological interventions. | ☐ | ☐ | ☐ | ☐ | ☐ | ☐ |
