# Supplementary material for: Knowledge, attitudes, and practice of general practitioners toward community detection and management of mild cognitive impairment: a cross-sectional study in Shanghai, China
Source: BMC Prim Care. 2022 May 11;23:114. doi: 10.1186/s12875-022-01716-9 (PMC9092880; doi:10.1186/s12875-022-01716-9)
Supplement: Supplementary file 3 — Additional file 3: Appendix File 3. Mediation regression results of predictors of MCI knowledge on practice scores via attitudes. [file 12875_2022_1716_MOESM3_ESM.docx]

**Appendix File 3. Mediation regression results of predictors of MCI knowledge on practice scores via attitudes**

| **Variable** | **Effect of knowledge on practice** | | | |  | **Effect of knowledge on attitudes** | | | |  | **Effect of knowledge and attitudes on practice** | | | |
| --- | --- | --- | --- | --- | --- | --- | --- | --- | --- | --- | --- | --- | --- | --- |
|  | **B** | **Beta** | ***t*** | ***p*** |  | **B** | **Beta** | ***t*** | ***p*** |  | **B** | **Beta** | ***t*** | ***p*** |
| Age (reference: <30 years) | | | | |  |  |  |  |  |  |  |  |  |  |
| 30-39 | -6.316 | 2.562 | -2.465 | 0.014 |  | -1.224 | 0.968 | -1.265 | 0.206 |  | -5.824 | 2.535 | -2.297 | 0.022 |
| 40-49 | -4.073 | 2.985 | -1.365 | 0.173 |  | 0.278 | 1.127 | 0.247 | 0.805 |  | -4.185 | 2.951 | -1.418 | 0.157 |
| ≥50 | -4.381 | 3.507 | -1.249 | 0.212 |  | -0.537 | 1.325 | -0.405 | 0.685 |  | -4.165 | 3.469 | -1.201 | 0.230 |
| Female (vs male) | 0.491 | 1.166 | 0.422 | 0.673 |  | 1.761 | 0.440 | 4.000 | **<0.001** |  | -0.216 | 1.160 | -0.186 | 0.852 |
| Unmarried (vs married) | 1.629 | 1.617 | 1.008 | 0.314 |  | -0.240 | 0.611 | -0.393 | 0.694 |  | 1.726 | 1.599 | 1.079 | 0.281 |
| Education (reference: <bachelor) |  |  |  |  |  |  |  |  |  |  |  |  |  |  |
| Bachelor | 0.254 | 2.878 | 0.088 | 0.929 |  | -2.311 | 1.087 | -1.127 | **0.034** |  | 1.182 | 2.850 | 0.415 | 0.678 |
| Postgraduate | 0.964 | 3.203 | 0.301 | 0.764 |  | -3.487 | 1.210 | -2.882 | **0.004** |  | 2.364 | 3.178 | 0.744 | 0.457 |
| Other Department (vs general practice) | 0.428 | 2.268 | 0.189 | 0.850 |  | 0.980 | 0.857 | 1.144 | 0.253 |  | 0.034 | 2.243 | 0.015 | 0.988 |
| Experience (reference:<5 years) |  |  |  |  |  |  |  |  |  |  |  |  |  |  |
| 5-9 | -0.785 | 1.955 | -0.402 | 0.688 |  | -1.179 | 0.738 | -1.597 | 0.111 |  | -0.311 | 1.935 | -0.161 | 0.872 |
| 10-14 | 1.845 | 2.103 | 0.879 | 0.380 |  | -1.883 | 0.794 | -2.371 | **0.018** |  | 2.604 | 2.084 | 1.249 | 0.212 |
| ≥15 | 1.300 | 2.387 | 0.545 | 0.586 |  | -2221 | 0.901 | -2.464 | **0.014** |  | 2.192 | 2.366 | 0.927 | 0.354 |
| Professional title (reference: primary) |  |  |  |  |  |  |  |  |  |  |  |  |  |  |
| Middle | -0.199 | 2.185 | -0.091 | 0.928 |  | -0.241 | 0.825 | -0.291 | 0.771 |  | -0.102 | 2.161 | -0.047 | 0.962 |
| Senior | -2.277 | 2.653 | -0.858 | 0.391 |  | 1.079 | 1.002 | 1.077 | 0.282 |  | -2.710 | 2.625 | -1.033 | 0.302 |
| Income (reference: <8000 Yuan) |  |  |  |  |  |  |  |  |  |  |  |  |  |  |
| 8000-11999 | -1.737 | 1.237 | -1.404 | 0.161 |  | 1.544 | 0.467 | 3.305 | **0.001** |  | -2.357 | 1.229 | -1.918 | 0.055 |
| ≥12000 | 1.395 | 1.719 | 0.812 | 0.417 |  | 2.092 | 0.649 | 3.222 | **0.001** |  | 0.555 | 1.707 | 0.325 | 0.745 |
| Daily visits (reference: <80) |  |  |  |  |  |  |  |  |  |  |  |  |  |  |
| 80-99 | -0.202 | 1.333 | -0.152 | 0.879 |  | -0.119 | 0.503 | -0.236 | 0.814 |  | -0.154 | 1.318 | -0.117 | 0.907 |
| ≥100 | -1.943 | 1.796 | -1.082 | 0.279 |  | 0.011 | 0.678 | 0.017 | 0.987 |  | -1.947 | 1.776 | -1.096 | 0.273 |
| MCI training (vs: without) | 2.775 | 1.285 | 2.159 | **0.031** |  | -0.241 | 0.485 | -0.496 | 0.620 |  | 2.872 | 1.271 | 2.259 | **0.024** |
| MCI screening qualification (vs: without) | 3.756 | 3.139 | 1.197 | 0.232 |  | -1.882 | 1.075 | -1.752 | 0.080 |  | 2.875 | 2.816 | 1.021 | 0.308 |
| The proportion of patients with memory disorder last month (reference: unsure) | | | | | | | | | |  |  |  |  |  |
| Zero | 3.756 | 3.139 | 1.197 | 0.232 |  | 1.954 | 1.186 | 1.649 | 0.099 |  | 2.971 | 3.107 | 0.956 | 0.339 |
| <10% | 3.808 | 2.310 | 1.649 | 0.099 |  | 0.321 | 0.872 | 0.368 | 0.713 |  | 3.679 | 2.283 | 1.611 | 0.107 |
| 10-29% | 3.029 | 2.411 | 1.256 | 0.209 |  | 0.794 | 0.911 | 0.872 | 0.384 |  | 2.710 | 2.385 | 1.136 | 0.256 |
| ≥30% | 7.931 | 3.135 | 2.530 | **0.012** |  | 2.003 | 1.184 | 1.692 | 0.091 |  | 7.127 | 3.103 | 2.296 | 0.022 |
| The proportion of patients with psychiatric symptoms last month (reference: unsure) | | | | | | | | | |  |  |  |  |  |
| Zero | -0.799 | 2.862 | -0.279 | 0.780 |  | -0.262 | 1.081 | -0.242 | 0.083 |  | -0.695 | 2.831 | -0.245 | 0.806 |
| <10% | -0.064 | 2.325 | -0.028 | 0.978 |  | 1.524 | 0.878 | 1.736 | 0.083 |  | -0.677 | 2.302 | -0.294 | 0.769 |
| 10-29% | 1.372 | 2.725 | 0.504 | 0.615 |  | 0.421 | 1.029 | 0.409 | 0.682 |  | 1.203 | 2.695 | 0.446 | 0.655 |
| ≥30% | 6.685 | 4.308 | 1.552 | 0.121 |  | -0.634 | 1.627 | -0.389 | 0.697 |  | 6.939 | 4.260 | 1.628 | 0.104 |
| MCI detection and management experience (vs: no/unsure) | 12.313 | 1.605 | 7.673 | **<0.001** |  | 1.511 | 0.606 | 2.493 | **0.013** |  | 11.706 | 1.591 | 7.358 | **<0.001** |
| Knowledge score | 0.178 | 0.031 | 5.696 | **<0.001** |  | 0.087 | 0.012 | 7.388 | **<0.001** |  | 0.143 | 0.032 | 4.527 | **<0.001** |
| Attitude score |  |  |  |  |  |  |  |  |  |  | 0.402 | 0.075 | 5.365 | **<0.001** |
| Adjusted R^2^ | 0.138 |  |  |  |  | 0.128 |  |  |  |  | 0.158 |  |  |  |

| **Effect of knowledge on practice** | **Effect** | **Se** | ***t*** | ***p*** | **95% CI** (**BC^a^)** |
| --- | --- | --- | --- | --- | --- |
| Total effect | 0.178 | 0.031 | 5.696 | <0.001 | 0.117-0.240 |
| Direct effect | 0.143 | 0.032 | 4.527 | <0.001 | 0.081-0.205 |
| Indirect effect | 0.035 | 0.008 |  |  | 0.019-0.052 |
| Partially standardized indirect effect(s) | 0.002 | <0.001 |  |  | 0.001-0.003 |
| Completely standardized indirect effect(s) | 0.032 | 0.007 |  |  | 0.018-0.048 |
